# Hemodynamic effects of Vernakalant in cardio-surgical ICU-patients treated for recent-onset postoperative atrial fibrillation

**DOI:** 10.1038/s41598-020-64001-8

**Published:** 2020-04-22

**Authors:** S. Schnaubelt, J. Niederdöckl, A. Simon, N. Schütz, C. Holaubek, M. Edlinger-Stanger, A. Niessner, B. Steinlechner, P. Sulzgruber, A. O. Spiel, H. Domanovits

**Affiliations:** 1 Department of Emergency Medicine, Medical University of Vienna, Vienna, Austria; 2 Division of Cardiothoracic and Vascular Anaesthesia and Intensive Care Medicine, Department of Anaesthesia, Intensive Care Medicine and Pain Medicine, Medical University of Vienna, Vienna, Austria; 3 Division of Cardiology, Department of Internal Medicine II, Medical University of Vienna, Vienna, Austria

**Keywords:** Cardiology, Drug therapy

## Abstract

Postoperative atrial fibrillation (POAF) is one of the most frequent complications after cardiothoracic surgery and a predictor for postoperative mortality and prolonged ICU-stay. Current guidelines suggest the multi-channel inhibitor Vernakalant as a treatment option for rhythm control. However, rare cases of severe hypotension and cardiogenic shock following drug administration have been reported. To elucidate the impact of Vernakalant on hemodynamics, we included ten ICU patients developing POAF after elective cardiac surgery, all of them awake and breathing spontaneously, in this prospective trial. Patients received the recommended dosage of Vernakalant and were clinically observed and monitored (heart rate, invasive blood pressure, pulse oximetry, central venous pressure) in 1-minute-intervals for 20 minutes before- and 120 minutes after the first dose of Vernakalant. The median time from the end of surgery until occurrence of POAF amounted up to 52.8 [45.9–77.4] hours, it took 3.5 [1.2–10.1] hours from occurrence of POAF until the first application of Vernakalant. All patients received catecholamine support with epinephrine that was held steady and not dynamic throughout the observational phase. We noted stable hemodynamic conditions, with a trend towards a reduction in heart rate throughout the 120 minutes after drug administration. In 7 patients (70%), conversion to sustained sinus rhythm (SR) occurred within 8.0 minutes [6.0–9.0]. No serious adverse events (SAEs) were noted during the observation period. In this prospective trial in ICU-patients showing POAF after cardiac surgery, intravenous Vernakalant did not induce clinically relevant negative effects on patients’ hemodynamics but resulted in conversion to sustained SR after a median of 8.0 minutes in 7 out of ten patients.

## Introduction

Postoperative atrial fibrillation (POAF) typically occurs around the second postoperative day and shows an especially high incidence of 40–60% in individuals undergoing cardio-surgical procedures. It represents a strong contributor for major cardiac adverse events in the short and the long run^1^. Recent evidence shows that POAF is linked to one third of postoperative strokes^1^. Moreover, POAF leads to complications such as ventricular arrythmias, congestive heart failure, the need for permanent pacemaker implantation, prolonged mechanical ventilation and ICU-stay, an increased risk for infection^1,2^, and is even associated with increased overall and cardiovascular mortality^3^. Various factors such as local inflammation, sympathetic activation or electrolyte imbalances have been discussed as etiologic factors for POAF development^4,5^. Whereas in severely symptomatic or hemodynamically unstable patients SR restoration by electrical cardioversion is persecuted, early pharmacologic cardioversion^6–8^ to prevent complications seems reasonable in the remaining patients, although POAF is often transient and self-limiting^9^. Hence, the relatively atrial selective and rate-dependent multi-channel-inhibitor Vernakalant has been recommended for cardioversion of POAF by current guidelines of the European Society of Cardiology (ESC)^3,10^. A beneficial efficacy and safety profile has been reflected in clinical studies^11–13^. However, – in contrast to the situation in Europe – the United States Food and Drug Administration (FDA) has not yet approved Vernakalant due to a case of severe hypotension and cardiogenic shock in the ACT V safety profiling study^14–16^. Thus, there is a need for further investigation of the safety profile of Vernakalant, especially in terms of hemodynamic changes after drug administration.

Therefore, our study objective was to observe the influence of Vernakalant on cardio-surgical patients’ hemodynamics and provide a descriptive report. Our studied group consisting of patients with chronic heart failure is of special interest concerning the hemodynamic drug response. We aimed at providing a stepping-stone for further research in large patient collectives.

## Methods

The study protocol was approved by the independent local Ethics Committee of the Medical University of Vienna (EC-No. 1161/2017). The trial was conducted according to the ICH-GCP guidelines (International Conference on Harmonization – Good Clinical Practice), as well as the Declaration of Helsinki, and meets the STROBE criteria. All subjects gave their written informed consent before inclusion. There was no contribution from the pharmaceutical industry at any stage of the trial.

### Study design

**Figure 1:**
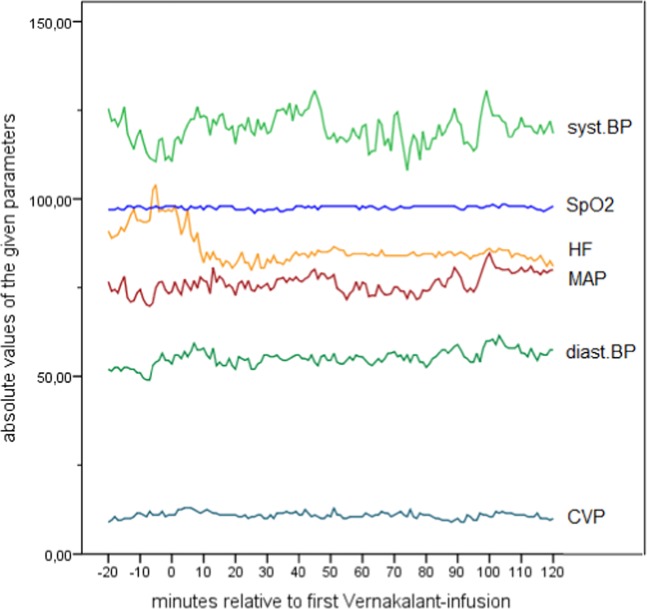
Median hemodynamic parameters of all included patients relative to the time point of the first Vernakalant-infusion (=0). syst.BP = systolic blood pressure in millimeters mercury, SpO2 = oxygen saturation in %, HF = heart rate in beats per minute, MAP = mean arterial pressure in millimeters mercury, diast.BP = diastolic blood pressure in millimeters mercury, CVP = central venous pressure in millimeters mercury.

Patients over 18 years of age being admitted to the cardiothoracic surgical intensive care unit (ICU) of the Medical University of Vienna, Austria, having undergone elective cardiac surgery (valve reconstruction/-replacement and/or coronary artery bypass grafting), and showing recent onset (<48 h) post-operative atrial fibrillation (POAF) were eligible for study inclusion screening. All included patients suffered from a form of ischemic heart failure (heart failure with reduced ejection fraction HFrEF, -mid-range ejection fraction HFmrEF, or -preserved ejection fraction HFpEF)^17^. Atrial fibrillation (AF) could be previously known or first documented, however, there must not have been an episode of AF until a minimum of 4 weeks before surgery. Patients were assigned to receive Vernakalant as part of their standard treatment plan. Prior to inclusion, several routine baseline and screening examinations were performed, including medical history and a standard 12-lead-electrocardiogram (ECG). Data from previously performed echocardiography was screened. The contraindications of intravenous Vernakalant – as provided by the manufacturer – were strictly followed and reviewed again by the study fellow before drug administration (Table 1). All patients received postoperative catecholamine treatment; no treatment changes were conducted during the study’s observational period. We did not include patients with atrial flutter as Vernakalant is not indicated in this patient group due to lack of efficacy^18^. By revision of arterial blood gas samples prior to inclusion, normal range potassium and magnesium levels were ensured or rebalanced as previously described elsewhere^19^. Vernakalant was administered by the treating physician intravenously as instructed by the manufacturer: up to two infusions (if POAF persisted) of Vernakalant (3 mg/kg the first and the second 2 mg/kg) in 100 ml of normal saline with an interruption of 15 minutes in between. The maximum amount of 339 mg for the first, and 226 mg for the second infusion was not exceeded^20^. The study’s main objective was to observe the influence of Vernakalant on patients’ hemodynamics. In this regard, patients were clinically observed and continuously monitored for 20 minutes before- and at least 2 h after administration of the first infusion. Monitoring included heart rate (HR), pulse oximetry (SpO2), central venous pressure (CVD) and invasive arterial blood pressure (iBP) (Fig. 1). Data points were collected in 1-minute-intervals. A median delta in blood pressure of 10 mmHg between before and after the administration of Vernakalant was chosen to be the cut-off for a clinically significant change.

**Table 1:** Contraindications for Vernakalant as provided by the manufacturer^20^. NYHA = New York Heart Association, ACS = acute coronary syndrome.

Contraindications for Vernakalant as provided by the manufacturer
• known allergies/hypersensitivities to Vernakalant hydrochloride or any other ingredients
• severe aortic stenosis
• systolic blood pressure <100 mmHg
• heart failure class NYHA III and IV
• prolonged QT (uncorrected > 440 msec)
• severe bradycardia, sinus node dysfunction or AV-block II°/III° without a pacemaker
• use of antiarrhythmic drugs (class I and III) 4 h prior to administration
• ACS 30 days prior to administration

According to the ICH-GCP guidelines, a serious adverse event (SAE) was defined as an event resulting in death, is a life-threatening condition, causes prolongation of existing hospitalization, results in persistent or significant disability/incapacity or requires intervention to prevent permanent impairment or damage.

### Outcome measurements

Deviations in hemodynamics measured from the baseline at 20 minutes prior to the first infusion of Vernakalant were defined as the primary endpoint. Conversion to SR was defined as the secondary-, the occurrence of adverse events as the tertiary endpoint.

### Statistical analysis

The study was conducted as an, observational pilot trial. Baseline and demographic data were tabulated. Data were presented as median and interquartile range (IQR) for continuous data and counts and percentages for discrete data. Chi-square test was used to assess the association of categorical data. Comparisons of continuous data between subgroups were performed using t-test. Statistical significance was defined by two-sided p-values of <0.05. Statistical analyses were performed using SPSS 21.0 (IBM SPSS, USA).

### Ethics approval and consent to participate

This study was approved by the local Ethics Committee (Ethics Committee of the Medical University of Vienna, Austria, EC-No. 1161/2017). Each patient gave oral and written informed consent for study inclusion and publication.

## Results

### Study population

From March 2017 to March 2018, a total of 24 patients were screened for eligibility. Fourteen were excluded due to another antiarrhythmic approach being favoured by the treating physician. Thus, ten patients were enrolled in this single-centre trial. The study population comprised of six (60%) male and four (40%) female patients with a median age of 76 (63–79) years, all of them awake and spontaneously breathing. Three patients had undergone valve reconstruction/-replacement with additional coronary artery bypass grafting, the other 7 patients had only received valve reconstruction/-replacement. Even though patients with HFmrEF and HFpEF were included, none suffered from heart failure class NYHA III or IV. None of the patients required respiration aids or oxygen insufflation at time of inclusion. Only 2 patients (20%) complained of POAF-related symptoms (thoracic pressure and vertigo). All participants were considered to be in a non-depleted volume status by the treating physician and showed sufficient urine output of >100 ml/h. Catecholamine support was received by all included patients (nor-epinephrine, 0.4 [0.2–0.7] mg/kg/minute), but not changed throughout the observational phase before and after the administration of Vernakalant (a systolic blood pressure of 85 mmHg was defined as the threshold of the necessity of an increase in catecholamine support).

The median time intervals from end of surgery until certain time points of interest amounted up to as follows: 52.8 h [45.9–77.4] until occurrence of POAF and 67.0 h [48.0–96.0] until the first drug infusion. The median time from occurrence of POAF until the first drug infusion was 3.5 h [1.2–10.1].

### Electrocardiographic and echocardiographic findings

No significant differences were noted in terms of electrocardiographic baseline values (e.g. prevalence of interatrial block). Echocardiography performed prior to study inclusion was screened: All patients suffered from chronic ischemic heart failure (6 patients with HFpEF with an ejection fraction of >50%, 3 patients with HFmrEF with an ejection fraction between 40 and 50% and one patient with HFrEF with an ejection fraction of <40%). There was no significant differences between the successful and unsuccessful cardioversion groups in left atrial size (45.6 ± 32.4 mm vs. 39.0 ± 33.8 mm, p = 0.791), ejection fraction measured by biplane Simpson method (49.2 ± 10.6% vs. 51.0 ± 1.4%, p = 0.727), diastolic function (all pseudo-normal pattern) or left ventricular diastolic diameter (60.0 ± 8.4 mm vs. 41.5 ± 2.1 mm, p = 0.627).

### Observational time and efficacy

In 7 patients (70%), conversion to sustained sinus rhythm (SR) could be achieved: in 6 cases AF converted after one infusion, in 1 patient a second infusion was necessary. The time until SR amounted to a median of 8.0 minutes [6.0–9.0].

### Safety

No SAEs were noted during the observational period. Minor adverse events comprised dysgeusia (n = 2; 20%), sneezing (n = 2; 20%), headaches (n = 1; 10%) and nausea without emesis (n = 1; 10%). These were self-limiting and did not require medical intervention.

### Effects of Vernakalant on hemodynamics

**Figure 2:**
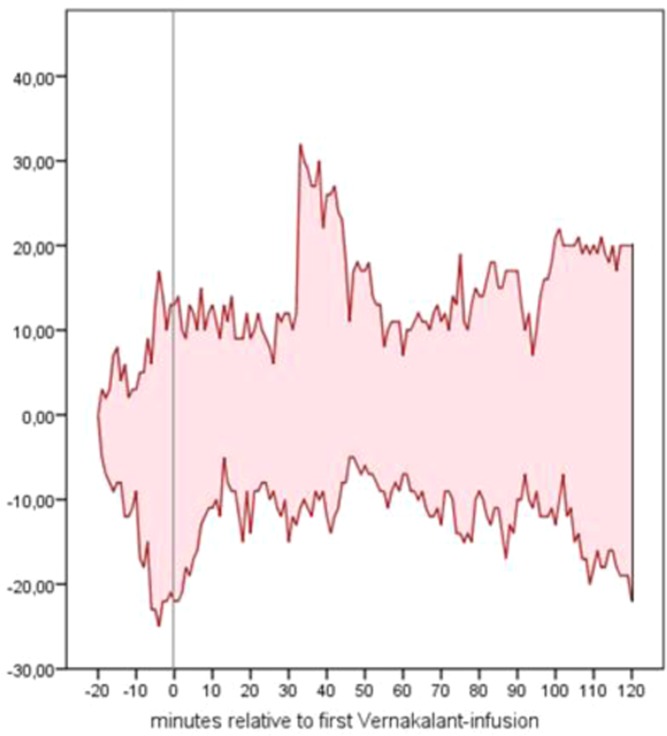
Range of dynamics in systolic blood pressure in millimeters mercury relative to the time point of the first Vernakalant-infusion (=0). The upper red line depicts the maximal delta of the systolic blood pressure compared to the baseline value; the lower red line depicts the minimal delta.

Patients’ baseline hemodynamics and the impact of Vernakalant is shown in Table 2. No significant changes could be noted. Recapitulating the range of blood pressure dynamics relative to the time point of the first Vernakalant infusion, a wide fluctuation of single values could be seen (Fig. 2). However, the minimal absolute MAP values that were recorded during the observational period ranged between 63 and 89 mmHg, therefore reflecting a clinically stabile blood pressure profile. Moreover and of utmost importance, no median delta in blood pressure values of over 10 mmHg - that was defined as clinically relevant beforehand - was seen. On the contrary, a trend towards a reduction in heart rate and positive stabilization in blood pressure values is observed.

**Table 2:** Baseline hemodynamics and the impact of Vernakalant. Pre-Vernakalant values were recorded before the first, post-Vernakalant values after the last admission. Data are given as medians [IQR], iBP = invasive blood pressure, bpm = beats per minute, mmHg = millimeters mercury.

Baseline hemodynamics and the impact of Vernakalant					
	baseline [IQR]	pre Vernakalant [IQR]	post Vernakalant [IQR]	mean difference [CI]	p-value
HR (bpm)	91.0 [84.3–101.8]	94.0 [84.6–101.3]	85 [72.5–89.3]	–11.8 [–28.7–5.1]	0.150
systolic iBP (mmHg)	125.5 [92.8–146.5]	116.5 [97.0–130.6]	121 [104.0–136.0]	5.6 [–1.9–13.1]	0.127
diastolic iBP (mmHg)	52.0 [48.0–64.8]	51.5 [49.5–61.8]	55.5 [51.5–59.0]	1.0 [–3.0–4.9]	0.596
MAP (mmHg)	77.0 [64.0–87.8]	73.6 [65.7–85.0]	75.5 [70.2–83.8]	2.5 [–1.9–7.0]	0.232
CVP (mmHg)	9.0 [8.8–10.8]	10.75 [9.8–13.0]	10.0 [9.8–12.3]	–0.6 [–1.7–0.6]	0.297
SpO2 (%)	97.0 [94.8–98.0]	97.8 [95.5–98.1]	98.0 [96.0–98.3]	0.2 [–0.6–0.9]	0.671

## Discussion

To our knowledge, this is the first study to continuously monitor HR, iBP, CVP and SpO2 at minimal intervals before, during and after administration of intravenous Vernakalant in ICU patients. Even though the oscillometric sphyngomanometric blood pressure measurement is widespread, there have been concerns of this method not being accurate in AF patients^21^, thus rendering the invasive measurement more reliable to offer precise results.

### Favourable efficacy

Times-to-conversion and efficacy data in our patient collective were comparable to previous results from our site^19^, whereas international efficacy data on Vernakalant is more variable, with a majority reporting conversion rates of around 50%^19,22^ and only sporadic reports of rates up to 70% and more^19,23^. As reported previously^18^, superior efficacy rates potentially result from a shorter AF-duration prior to conversion (in our population a median of 3.5 h). Comparing our results with the two subgroups of POAF- and ICU patients, a superior performance of Vernakalant in our collective can be noted (e.g. a median of 12.4 minutes and a response-rate of 44.9% in 107 POAF-patients in 2015^12^, or a median of 30 minutes and a response-rate of 53% in an ICU-population of 32 patients in 2014^24^).

There was a median drop in HR of 11.8 bpm, reflecting the high potency of Vernakalant to restore normofrequent SR.

### A real-life safety profile and a tendency towards blood pressure stabilization

No proarrhythmic effect of Vernakalant could be observed in our study collective, probably due to the relative atrial selectivity of the drug^25,26^. Minor adverse events including sneezing, dysgeusia and nausea, were lying within the known side effect profile of Vernakalant that is caused by inhibition of sodium-channels in the central nervous system^19,27^.

Typical episodes of hypotension after administrating Vernakalant occur during or immediately after the infusion and are usually transient and fluid-responsive^12,16,28^. Moreover, a general slight decrease in blood pressure lasting several hours after administration of Vernakalant and rare cases of severe events of hypotension and cardiac shock have been published^12,14,19,24^. Whereas short BP-drops during and immediately after cardioversion could be an expression of atrial stunning and therefore reduced cardiac output^29,30^, a long-lasting reduction in BP could be mediated through reduced sympathetic impulses or reduced activation of the RAAS^31^.

However, Vernakalant appeared to have likewise the potential to increase systolic and diastolic blood pressure in euvolemic patients during the first 20–40 minutes after the infusion^19^. This effect was also noted in our study collective: an overall increase of systolic iBP of 5.6 mmHg was achieved during the 2h-observational-period after having administered Vernakalant. Although conflicting results about how and when exactly restoration of SR can increase BP-values, one theory is the atrial contractility being increased and therefore a greater ejection fraction resulting from the left ventricle^32^. Comparing Vernakalant to other antiarrhythmic agents such as Ibutilide^19^, the slight and immediate effect of BP-increase could be a distinct feature of Vernakalant and not just related to the cardioversion itself. Further research into this topic should be conducted in the future to clarify this open question.

No differences could be seen comparing pre-Vernakalant echocardiography between the successful and unsuccessful cardioversion group; this may result from the small sample size.

No notable change in CVP before and after the administration of Vernakalant could be noted. With increased CVP being an indirect marker for increased global ventricular preload^33,34^, and our study collective remaining at constant CVP-levels, at least a situation of non-inferiority in terms of patients’ volume status after being treated with Vernakalant can be suggested.

### Future applications

Results from the EAST-trial - hopefully giving further insight to the potential benefit of early rhythm control therapy - are still pending^9^, and rhythm control could in the future be regarded as superior to rate control, leading to a significant increase in cardioversions undertaken at Emergency Departments and (postoperative) ICUs. Clinicians need a reliable and safe tool for quick cardioversion and Vernakalant is in this context a very promising drug. In our study collective no relevant changes in hemodynamics, especially the invasive measured blood pressure was detected. The favorable safety profile of Vernakalant is therefore supported if the manufacturer’s contraindications are strictly applied and Vernakalant is administered to euvolemic patients. Further validation of our results within a larger patient collective is recommended.

### Limitations

Our study has several limitations. The small sample size can only give an impulse for further research and cannot translate into general treatment recommendations. There was no comparison- or placebo subgroup to further validate our results. Lastly, our single-centre approach potentially biased results due to local standards of care affecting outcomes.

## Conclusion

In this prospective trial in ICU-patients showing POAF after cardiovascular surgery, intravenous Vernakalant did not induce clinically relevant negative effects on the patients’ hemodynamics. Moreover, a high rate of conversion to sustained SR after a median of 8.0 minutes was observed. Therefore, within the boundaries of known contraindications, the use of Vernakalant should be considered in euvolemic, hemodynamic stable ICU patients with POAF. Given the limited sample size, further research is needed to confirm our findings.

## Supplementary information

STROBE checklist.

## Data Availability

The datasets acquired and/or analyzed during the current study are available from the corresponding author on reasonable request.
